# Association of 24-hour movement guideline adherence with inhibitory control in older adults: the mediating role of general self-efficacy

**DOI:** 10.3389/fpsyg.2026.1872136

**Published:** 2026-07-22

**Authors:** Zhiji Wang, Li Zhan, Sijun Wu, Shijie Liu, Chicheng Zhou, Dandan Wang, Hong Wang, Lin Wang

**Affiliations:** 1 School of Physical Education, Wuhan University of Technology, Wuhan, China; 2 Center for Hubei Ethnic Traditional Sports Culture Preservation and Innovation, Wuhan, China; 3 School of Physical Education, Shanghai University of Sport, Shanghai, China; 4 School of Physical Education, Wuhan University of Bioengineering, Wuhan, China; 5 School of Wushu, Wuhan Sport University, Wuhan, China

**Keywords:** 24-h movement guidelines, general self-efficacy, inhibitory control, mediation effect, older adults

## Abstract

**Objective:**

In the context of population aging, inhibitory control is important for maintaining independent living, behavioral regulation, and healthy aging in later life. Although physical activity, sedentary behavior, sleep, and general self-efficacy have all been linked to cognitive health, direct evidence remains limited regarding whether older adults’ alignment with integrated 24-h movement behavior recommendations relates to inhibitory control and whether psychological resources contribute to this relationship. From a 24-h movement behavior perspective, the present study evaluated the link between the number of recommendations met and Stroop-indexed inhibitory control in older adults and tested the mediating role of general self-efficacy.

**Methods:**

This cross-sectional survey analyzed data from 210 older adults in four Wuhan communities. Accelerometer recordings were used to capture 24-h movement behaviors, and recommendation status was determined based on physical activity, sedentary behavior, and sleep criteria. Participants completed the validated Chinese version of the General Self-Efficacy Scale, and inhibitory control was measured using an E-Prime-based Stroop task. A structural equation model estimated the direct and indirect pathways, with relevant covariates controlled.

**Results:**

Among the 210 older adults, a greater number of recommendations met corresponded to higher general self-efficacy (*β* = 0.209, 95% CI [0.067, 0.343]) and better inhibitory control performance (*β* = 0.240, 95% CI [0.112, 0.369]). General self-efficacy was also positively linked to inhibitory control (*β* = 0.188, 95% CI [0.059, 0.306]). The pathway operating through general self-efficacy reached statistical significance (*β* = 0.039, 95% CI [0.009, 0.092]) and represented 14.1% of the total effect.

**Conclusion:**

Meeting more 24-h movement behavior recommendations was associated with better inhibitory control in older adults. General self-efficacy showed a small but statistically significant indirect association in this relationship, suggesting that it may partly account for the link between guideline adherence and inhibitory control, but cannot fully explain it. From a health promotion perspective, these findings highlight the potential value of integrated strategies that promote 24-h movement behavior guideline adherence and consider self-efficacy-related psychological resources to support cognitive and psychological health in later life.

## 1 Introduction

As populations age, preserving the cognitive abilities that support independent living has become a central concern in healthy aging research. Although dementia incidence has declined in some settings, longer life expectancy and the growing number of older adults mean that age-related cognitive decline and dementia-related disability remain major public health challenges (De Pue et al., 2025; Livingston et al., 2024). Within this context, executive function is particularly relevant because it enables older adults to organize behavior, maintain goals, adapt to changing situations, and participate in everyday activities (Dexter and Ossmy, 2023). Inhibitory control, one core component of executive function, refers to the capacity to withhold dominant responses, ignore task-irrelevant stimuli, and maintain attention on current goals. These processes are closely connected with daily self-regulation in older adults, including attentional control, health-related decision-making, behavioral inhibition, and fall-risk management. Previous evidence indicates that inhibitory control changes with age at both behavioral and neural levels, while compensatory neural recruitment may occur during cognitive control tasks (Kang et al., 2021). Because this cognitive domain may remain responsive to behavioral and psychological influences, identifying related factors could inform efforts to preserve cognitive health, functional independence, and quality of life in later life.

Research on movement behaviors has increasingly shifted from single-behavior models to an integrated 24-h perspective. Within this framework, physical activity (PA), sedentary behavior (SB), and sleep are viewed as interdependent components of the same finite day, rather than as isolated behaviors. For older adults, this perspective is especially relevant because lower PA, prolonged sedentary time, and changes in sleep patterns often occur together. Focusing on one behavior alone may miss how time allocated to one domain necessarily reduces time available for another, as well as how these behaviors combine to shape health-related outcomes (Ross et al., 2020). Evidence from older populations also suggests that overall adherence to 24-h movement behavior guidelines remains low, whereas meeting more recommendations is generally linked to more favorable physical and psychological health outcomes (Liang et al., 2024). Accordingly, a 24-h movement framework may provide a more realistic basis for understanding healthy aging than approaches that evaluate PA, SB, or sleep separately.

A growing body of evidence connects 24-h movement behaviors with executive function in older adults. A review involving more than 23,000 older adults indicated that higher PA, less passive SB, and appropriate sleep duration tend to co-occur with better cognitive performance (Mellow et al., 2022). In older samples, compositional analyses have further shown that the allocation of time across 24-h behaviors is related to executive function, with more time spent in moderate-to-vigorous PA or light PA being associated with more favorable cognitive outcomes, including inhibitory control (Hyodo et al., 2022; Marent et al., 2025). Evidence from randomized trials and research syntheses also indicates that exercise interventions can benefit executive function in middle-aged and older adults, with inhibitory control emerging as one of the more consistent domains of improvement (Martini et al., 2024; Ye et al., 2024). Collectively, these studies suggest that associations between movement behaviors and executive function in older adults may be domain-specific rather than uniform across all executive function subdomains. For example, one older-adult study examining both planning and problem-solving performance and Stroop-based inhibitory control showed that combined PA and SB patterns were not uniformly related to all executive function measures, highlighting that movement behavior–cognition associations may depend on the executive function domain being assessed (Chen et al., 2024). Meta-analytic and functional-connectivity evidence further indicates that age-related executive function changes are heterogeneous and may involve both domain-general and process-specific patterns, supporting the need to examine more narrowly defined subprocesses (Heckner et al., 2021). Inhibitory control is particularly relevant in this context, as evidence from Stroop paradigms indicates that cognitive inhibition is sensitive to later-life changes, and recent older-adult research has examined associations between habitual stepping activity and Stroop task performance (Forte et al., 2024; O'Brien et al., 2024). Therefore, focusing on inhibitory control provides a theoretically relevant basis for examining 24-h movement behavior guideline adherence in relation to a specific executive function domain in older adults. However, most existing studies have focused on time-use composition, single movement behaviors, or global executive performance. Less attention has been given to guideline adherence as a public health-oriented indicator, and direct evidence for inhibitory control as a specific executive subdomain remains limited. Therefore, behavioral time allocation alone may not fully explain individual differences in cognitive control in later life, and psychological mechanisms need to be considered to clarify this relationship.

Self-efficacy provides a psychological lens for explaining how older adults organize and sustain health-related behaviors in daily life. Within social cognitive theory, efficacy beliefs reflect individuals’ judgments about their ability to initiate actions, regulate behavior, and persist when difficulties arise. General self-efficacy extends beyond confidence in a specific task or behavior and represents a broader appraisal of one’s capacity to cope with challenges across situations (Whitehall et al., 2021). These beliefs are shaped by accumulated mastery experiences, physiological and emotional states, and repeated evaluations of whether one’s actions lead to desired outcomes (Young et al., 2014). This perspective is particularly relevant to 24-h movement behaviors because PA, SB, and sleep all require daily regulation. In middle-aged and older adults, self-efficacy has been related to outcome expectations, goal setting, and actual activity engagement (Mcmahon et al., 2024; Yu et al., 2025). Older adults with stronger exercise-related self-efficacy tend to report higher PA and less SB, whereas poorer sleep quality has been observed among those with lower self-efficacy in community samples (Szczuka et al., 2021; Wu et al., 2026; Xie et al., 2025). Taken together, this evidence suggests that general self-efficacy may be closely tied to older adults’ capacity to maintain more favorable daily movement and recovery patterns.

For older adults, meeting more 24-h movement recommendations may indicate a daily routine that is more structured, active, and sustainable. Such a routine can provide repeated experiences of successfully maintaining health-related behaviors, while lower SB, more appropriate sleep, and better perceived physical states may strengthen older adults’ sense of control over daily life (Whitehall et al., 2021). Although much of the available evidence concerns specific efficacy beliefs, such as exercise self-efficacy or cognitive self-efficacy, it nonetheless suggests that older adults who feel more capable of managing daily activities, regulating physical and psychological states, and persisting toward goals are more likely to report stronger efficacy-related beliefs (Mcmahon et al., 2024; Su et al., 2026). These beliefs may also matter in cognitive task contexts. Older adults with stronger self-efficacy may be more willing to invest effort, maintain task goals, and use control strategies when facing cognitive conflict. Consistent with this view, higher self-efficacy has been related to better cognitive performance in older samples, and improvements in cognitive self-efficacy have occurred alongside gains in cognitive function (Abdelsalam and Elkholy, 2024; Suyasith et al., 2025). General self-efficacy may therefore help characterize the psychological resources that are associated with healthier 24-h movement patterns and better inhibitory control.

Overall, research on 24-h movement behaviors has developed rapidly, but the available evidence remains heterogeneous in terms of study populations, behavioral assessment methods, and statistical approaches. Findings across different populations and health outcomes therefore still require further integration. In older adults, evidence is still limited on whether meeting more 24-h movement behavior recommendations relates specifically to inhibitory control, and whether general self-efficacy contributes indirectly to this relationship. Based on an integrated 24-h movement behavior perspective, the present study investigated the relationship between meeting more guideline recommendations and inhibitory control in older adults, and further tested the mediating role of general self-efficacy. By doing so, this study aimed to provide new theoretical evidence for understanding the psychological mechanism linking daily movement behaviors with cognitive control in later life.

Accordingly, the present study addressed the following hypotheses:

*H1*: Meeting more 24-hour movement behavior recommendations would be positively related to inhibitory control in older adults.

*H2*: General self-efficacy would mediate the relationship between meeting more 24-hour movement behavior recommendations and inhibitory control in older adults.

## 2 Materials and methods

### 2.1 Sample size estimation

The target sample size was estimated before recruitment using Monte Carlo power analysis for indirect effects, which matched the cross-sectional mediation framework of this study. The calculation was performed using the web-based Monte Carlo Power Analysis for Indirect Effects application (Schoemann et al., 2017), which is designed for estimating power and sample size in simple and complex mediation models.^1^ The confidence level was set at 95%, the desired statistical power at 0.80, the number of replications at 2,000, and the number of Monte Carlo draws per replication at 20,000. The calculation suggested that a minimum of 169 participants would provide 0.80 statistical power. Because incomplete questionnaires, unsuccessful inhibitory control testing, or invalid accelerometer data were possible among older participants, recruitment was planned above this minimum requirement.

### 2.2 Participants

Participants were enrolled from September 2024 to June 2025 in four residential communities in Wuhan, Hubei Province, China: the East Community and West Community of the Mafangshan Campus of Wuhan University of Technology, Youli Community, and Jiangdayuan Community. Recruitment was carried out through posters and in-person health education lectures. In total, 237 older adults entered the initial recruitment pool.

The inclusion criteria were: (1) aged ≥65 years and residence in the current community for at least 6 months; (2) having basic communication ability, being able to understand the study procedures, and being able to complete the questionnaires and computerized cognitive tasks under the guidance of the researchers; (3) independently mobile and able to wear an accelerometer for 7 consecutive days as required; (4) able to complete the physical activity log during the monitoring period.

The exclusion criteria were: (1) a previous diagnosis of severe neurological or psychiatric disorders, including Alzheimer’s disease, epilepsy, Parkinson’s disease, or major psychiatric disorders; (2) severe visual, hearing, or language communication impairments; (3) severe limb dysfunction or mobility limitations that prevented accelerometer wear; (4) failure to complete the questionnaires, computerized cognitive task, or accelerometer monitoring as required.

Of the 237 participants initially recruited, 12 were excluded for not completing all study procedures, and 15 were excluded because of insufficient accelerometer wear time or invalid accelerometer data. The final analysis therefore included 210 older adults with complete and valid data, which met the minimum sample size requirement. This study was approved by the Scientific Research Ethics Committee of Wuhan Sport University (Approval No. 2025119), and all participants provided written informed consent before participation.

### 2.3 Measures

#### 2.3.1 24-h movement behaviors

The 24-h movement behaviors were objectively monitored using an ActiGraph wGT3X-BT accelerometer, which has been widely applied in studies assessing PA, SB, and sleep (Full et al., 2018; Sasaki et al., 2011). Participants were instructed to place the device on the wrist of the non-dominant hand throughout the monitoring period, except when removal was necessary, such as during bathing or swimming. Data were recorded in 60-s epochs. Monitoring began at 12:00 on the day the accelerometer was distributed and continued until 12:00 on day 8, providing 7 consecutive days of data, including five weekdays and two weekend days. The device was collected by the research team on the eighth day.

After monitoring, accelerometer data were processed in ActiLife 6.13.3. Movement intensity was classified according to the cut-off points: 0–99 counts/min represented SB, 100–1,951 counts/min represented light-intensity physical activity (LPA), and >1,951 counts/min represented moderate-to-vigorous physical activity (MVPA) (Freedson et al., 1998; Miller et al., 2010). Although a limited number of placement-specific ActiGraph cut-points have been proposed for adults and older adults, no universally accepted set of wrist-specific cut-points for older adults has been established. Therefore, the conventional ActiGraph count-based cut-points were retained to maintain consistency with previous count-based adult and older-adult studies and to facilitate comparison with guideline-adherence research using similar PA and SB indicators (Bammann et al., 2021; Migueles et al., 2021). A day was treated as valid when accelerometer wear time reached at least 10 h. Participants were retained in the analysis when they contributed at least three valid monitoring days (2 weekdays and 1 weekend day). A paper-based activity log was also distributed with the accelerometer. Participants recorded their daily activities, sleep onset, and wake times, and these records were used to check and calibrate the accelerometer-derived data.

Guideline adherence was assessed across three behavioral domains: PA, SB, and sleep. PA adherence was determined from accelerometer-derived MVPA duration. For participants with valid data for all 7 monitoring days, weekly MVPA was calculated by summing MVPA time across the full monitoring period. For those who met the valid monitoring criteria but did not have complete 7-day data, weekly MVPA was estimated as follows: mean weekday MVPA × 5 + mean weekend-day MVPA × 2. Participants with weekly MVPA ≥150 min were identified as meeting the PA recommendation (Bull et al., 2020; Ross et al., 2020).

Sleep duration was determined using continuous accelerometer data together with the activity logs. Two researchers used the recorded sleep onset and wake times to assist in identifying nighttime sleep duration for each valid monitoring day. The mean nighttime sleep duration across all valid days was used as each participant’s average daily sleep duration. Participants with an average daily sleep duration of 7–8 h were identified as meeting the sleep recommendation (Ross et al., 2020; Šuc et al., 2024).

SB was assessed from accelerometer-derived sedentary time. Mean daily sedentary time was calculated across all valid monitoring days, and participants with mean sedentary time ≤8 h/day were identified as meeting the SB recommendation (Rojer et al., 2021; Ross et al., 2020). The total number of recommendations met was calculated by summing adherence across PA, SB, and sleep. Scores ranged from 0 to 3, with higher scores indicating adherence to more dimensions of the 24-h movement behavior guidelines. The adherence count score was used as the primary exposure variable because it provides a simple and public health-oriented indicator of whether older adults achieved a greater number of recommended movement behavior targets. This approach is consistent with guideline-based surveillance studies and allows the results to be interpreted in relation to practical behavioral recommendations.

#### 2.3.2 General self-efficacy

Participants completed the General Self-Efficacy Scale (GSES) to report their perceived coping capacity. The scale contains 10 items rated on a 4-point Likert scale (response options ranging from 1 = not at all true to 4 = exactly true) (Schwarzer and Renner, 2000). Responses were summed to obtain a total score; higher values reflected stronger general self-efficacy. The present study used the Chinese version of the GSES, which has been applied in older Chinese populations and has demonstrated acceptable reliability and validity (Ji-Liang and Dan, 2004). In the present sample, internal consistency was high, with a Cronbach’s *α* of 0.93.

#### 2.3.3 Inhibitory control

Inhibitory control was measured using a computerized Stroop task programmed in E-Prime 2.0.10.182. The task used four Chinese words, “蓝”(blue), “黄”(yellow), “红”(red), and “绿”(green), displayed in blue, yellow, red, or green font colors. Participants judged whether word meaning and font color were congruent. For congruent trials, in which the word meaning and font color matched, participants pressed the “F” key as quickly as possible. For incongruent trials, in which the word meaning and font color differed, participants pressed the “J” key.

Each trial began with a central fixation cross (500 ms), followed by a blank screen (1,000 ms). The stimulus was then displayed for 1,500 ms, during which participants had to respond. The task included 16 practice trials followed by 72 formal trials, consisting of 36 congruent and 36 incongruent trials. The formal trials were presented in a randomized order. Accuracy and reaction time were recorded for each trial and then summarized separately for congruent and incongruent trials. Trials with reaction times shorter than 200 ms were excluded from reaction-time calculation. Mean reaction time was calculated from correct-response trials, whereas accuracy was calculated as the proportion of correct responses across valid trials.

To address the speed–accuracy trade-off in Stroop performance, reaction time and accuracy were converted into a balanced integration score (BIS), which served as the dependent variable for inhibitory control (Liesefeld and Janczyk, 2019). Reaction time and accuracy were separately z-standardized across participants, and BIS was calculated by subtracting standardized reaction time from standardized accuracy, using the following formula:

BIS=z(ACC)−z(RT)

BIS was selected because it combines speed and accuracy into a single performance index and is therefore suitable for older-adult cognitive task data, in which slower but more accurate responses, or faster but less accurate responses, may otherwise complicate interpretation. Higher BIS values indicate better overall Stroop performance, reflecting relatively higher accuracy and/or faster responses.

### 2.4 Covariates

Demographic information covered sex, age, BMI, monthly income level, and educational level. Sex was categorized as female or male. Monthly income level was categorized as <5,000 RMB/month or ≥5,000 RMB/month. Educational level was categorized as primary school or below, secondary school, or undergraduate degree and above. BMI was computed as body weight divided by height squared (kg/m^2^).

### 2.5 Statistical analysis

Data were processed in SPSS 31.0 and AMOS 31.0. Descriptive analyses were first performed for the main variables and covariates. Continuous variables were summarized as mean ± standard deviation (M ± SD), and categorical variables as frequencies and percentages. Descriptive Pearson correlation analyses were conducted before structural equation modeling to summarize the bivariate relationships among the number of guideline recommendations met, general self-efficacy, inhibitory control, and covariates.

Before testing the mediation model, we checked the distribution of the main variables and examined potential multicollinearity among predictors. Skewness and kurtosis were calculated to evaluate whether the data were appropriate for parametric analyses. Variance inflation factors (VIFs) were calculated to check multicollinearity.

A structural equation modeling approach was then applied to test the hypothesized indirect pathway from meeting more guideline recommendations to inhibitory control through general self-efficacy. Model fit was evaluated using the chi-square to degrees of freedom ratio (χ^2^/df), goodness-of-fit index (GFI), incremental fit index (IFI), comparative fit index (CFI), root mean square error of approximation (RMSEA), and standardized root mean square residual (SRMR). Model fit was considered acceptable when χ^2^/df < 3, GFI, IFI, and CFI > 0.90, RMSEA < 0.08, and SRMR < 0.08.

The indirect pathway was evaluated with a bootstrap procedure using 5,000 resamples. Bias-corrected 95% confidence intervals (CIs) were estimated after adjustment for age, sex, BMI, educational level, and monthly income level. Evidence for an indirect effect was inferred when the 95% CI did not include zero. All statistical tests were two-tailed, and *p* < 0.05 was used as the threshold for statistical significance.

## 3 Results

A total of 210 older adults were included in the analysis (Table 1). The mean age was 70.60 years (SD = 6.17), and the mean BMI was 23.65 kg/m^2^ (SD = 2.74). The mean GSES score was 26.80 (SD = 5.79). For individual guideline components, 142 participants (67.6%) met the PA recommendation, 65 participants (31.0%) met the SB recommendation, and 38 participants (18.1%) met the sleep recommendation. For the number of guideline recommendations met, 23 participants (11.0%) met no recommendation, 129 (61.4%) met one recommendation, and 58 (27.6%) met two recommendations; none of the participants met all three recommendations. Stroop task performance (BIS) had a mean value of −1.59 (SD = 0.84). To further describe the distribution of selected variables across educational levels, subgroup descriptive statistics for age, sex, and the number of guideline recommendations met are presented in Supplementary Table S1.

**Table 1:** Characteristics of the study participants (*n* = 210).

Variables	Mean/frequency	SD/%
Age	70.60	6.17
Sex		
Male	85	40.5%
Female	125	59.5%
BMI	23.65	2.74
Educational level		
Primary school or below	25	11.9%
Secondary school	98	46.7%
Undergraduate degree or above	87	41.4%
Monthly income level		
<5,000	94	44.8%
≥5,000	116	55.2%
24-h movement behavior guideline adherence		
Meeting PA recommendation	142	67.6%
Meeting SB recommendation	65	31.0%
Meeting sleep recommendation	38	18.1%
Number of guideline recommendations met		
0	23	11.0%
1	129	61.4%
2	58	27.6%
3	0	0.0%
General self-efficacy	26.80	5.79
Inhibitory control		
Accuracy in congruent trials	0.86	0.12
Reaction time in congruent trials	912.88	134.30
Accuracy in incongruent trials	0.80	0.14
Reaction time in incongruent trials	1118.98	146.63
BIS	−1.59	0.84

SD, Standard deviation; reaction time is reported in milliseconds.

### 3.1 Normality and multicollinearity diagnostics

Before the correlation and path analyses, the distribution of the main variables and potential multicollinearity among predictors were checked. The number of guideline recommendations met, GSES score, and Stroop task performance showed skewness from −0.269 to −0.080 and kurtosis from −0.353 to 0.505, all within the commonly accepted range of ±2. These results suggested that the distributions of the main variables were suitable for subsequent parametric analyses. Regression diagnostics were then performed with Stroop task performance as the outcome variable and the number of guideline recommendations met, GSES score, and covariates as predictors. Tolerance ranged from 0.883 to 0.967, and VIF ranged from 1.035 to 1.132, indicating no marked multicollinearity among the predictors.

### 3.2 Correlations among the number of guidelines met, general self-efficacy, and inhibitory control

Correlation analyses identified positive relationships among variables (Table 2). The number of guideline recommendations met showed a positive correlation with GSES score (r = 0.211, *p* < 0.01) and Stroop task performance (r = 0.273, *p* < 0.001). GSES score was positively related to Stroop task performance (r = 0.250, *p* < 0.001). These results indicated that older adults who met more guideline recommendations tended to report stronger general self-efficacy and perform better on the Stroop task.

**Table 2:** Correlations among adherence to the 24-h movement behavior guidelines, general self-efficacy, and inhibitory control.

Variables	Age	Sex	BMI	Educational level	Monthly income level	GSES	Number of guideline recommendations met	BIS
Age								
Sex	0.132							
BMI	−0.119	−0.123						
Educational level	−0.120	−0.129	0.041					
Monthly income level	0.096	−0.040	0.065	0.082				
GSES	−0.147*	−0.046	−0.016	0.250***	0.103			
Number of guideline recommendations met	−0.068	−0.159*	−0.181**	0.008	0.075	0.211**		
BIS	0.045	0.083	−0.167*	0.129	0.021	0.250***	0.273***	

*p < 0.05, **p < 0.01, ***p < 0.001.

For the covariates, age was negatively correlated with GSES score (r = −0.147, *p* < 0.05), whereas educational level was positively related to GSES score (r = 0.250, *p* < 0.001). BMI showed a negative relationship with Stroop task performance (r = −0.167, *p* < 0.05). The number of guideline recommendations met was related to sex (r = −0.159, *p* < 0.05) and BMI (r = −0.181, *p* < 0.01), but not to age, educational level, or monthly income level.

### 3.3 Association between adherence to the 24-h movement behavior guidelines and inhibitory control in older adults and the indirect effect of general self-efficacy

After controlling for age, sex, BMI, educational level, and monthly income level, path analysis examined whether GSES score indirectly linked the number of guideline recommendations met with Stroop task performance. The model showed acceptable fit: χ^2^ (df = 6) = 11.676, χ^2^/df = 1.946, *p* = 0.070; GFI = 0.986; IFI = 0.939; CFI = 0.920; RMSEA = 0.067; and SRMR = 0.042. These results indicated that the model was adequately fitted to the data and could be used to interpret the subsequent path relationships.

**Figure 1:**
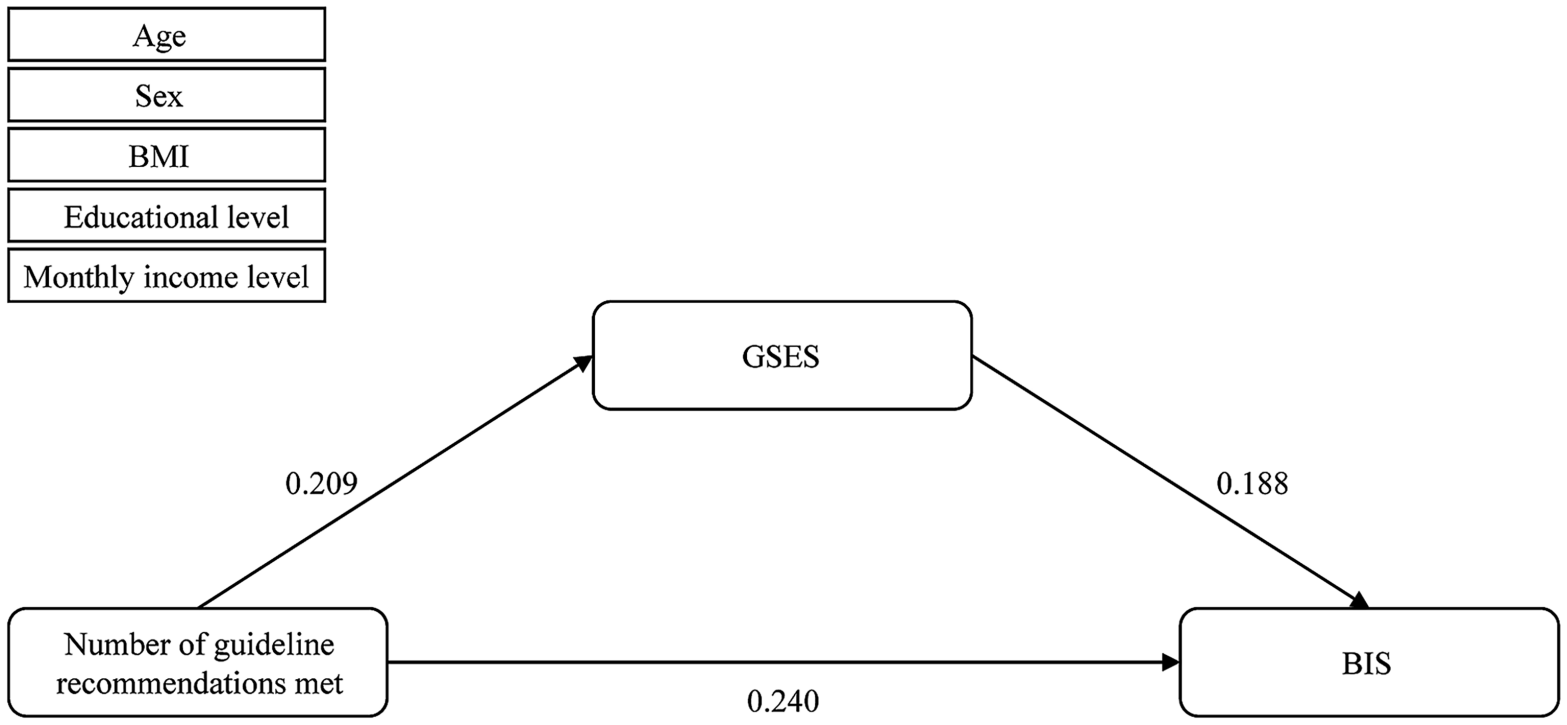
Mediation model of the association between adherence to the 24-h movement behavior guidelines and inhibitory control through general self-efficacy. Values on paths are standardized coefficients. The model was adjusted for age, sex, BMI, educational level, and monthly income level. GSES, General Self-Efficacy Scale; BIS, balanced integration score.

The mediation model examined the relationships among the number of guideline recommendations met, GSES score, and Stroop task performance. The direct path coefficients are presented in Table 3 and Figure 1. After adjustment for covariates, the number of guideline recommendations met showed a positive path to GSES score (*β* = 0.209, 95% CI [0.067, 0.343]) and Stroop task performance (*β* = 0.240, 95% CI [0.112, 0.369]). GSES score also showed a positive path to Stroop task performance (*β* = 0.188, 95% CI [0.059, 0.306]). These results indicated that older adults who met more guideline recommendations had higher GSES scores and better Stroop task performance.

**Table 3:** Direct path coefficients among adherence to the 24-h movement behavior guidelines, general self-efficacy, and inhibitory control.

Direct path	β	Lower	Upper
Number of guideline recommendations met — General self-efficacy	0.209	0.067	0.343
Number of guideline recommendations met — Inhibitory control	0.240	0.112	0.369
General self-efficacy — Inhibitory control	0.188	0.059	0.306

Bootstrap results indicated a significant indirect effect from the number of guideline recommendations met to Stroop task performance through GSES score (*β* = 0.039, 95% CI [0.009, 0.092]) (Table 4). This result indicated that GSES score mediated the relationship between the number of guideline recommendations met and Stroop task performance. Because the direct effect remained significant, GSES score showed a partial mediating effect. The indirect effect accounted for approximately 14.1% of the total effect. These results suggested that the number of guideline recommendations met was linked to Stroop task performance mainly through a direct pathway, with part of the association occurring indirectly through GSES score.

**Table 4:** Indirect effect of adherence to the 24-h movement behavior guidelines on inhibitory control through general self-efficacy.

Indirect effect	β	Lower	Upper
Number of guideline recommendations met — General self-efficacy — Inhibitory control	0.039	0.009	0.092

## 4 Discussion

The present findings connect meeting 24-h movement behavior guideline recommendations with general self-efficacy and inhibitory control in older adults. Participants who met more guideline recommendations tended to display better inhibitory control and to report higher general self-efficacy. Path analysis further showed a statistically significant but modest indirect association through general self-efficacy, suggesting that the association between meeting more guideline recommendations and inhibitory control remained primarily direct. Overall, these findings supported the hypotheses of the present study and indicated that behavioral patterns across the 24-h day and self-efficacy-related psychological resources may both be relevant to inhibitory control, although they may differ in strength and mode of influence.

First, inhibitory control was better among older adults who achieved a higher count of guideline recommendations. This finding can be interpreted within the integrated perspective of 24-h movement behaviors, in which PA, SB, and sleep are viewed as interdependent parts of the same day rather than isolated exposures. From this perspective, meeting more recommendations may reflect the co-occurrence of sufficient PA, limited sedentary time, and appropriate sleep duration, forming a more favorable overall daily behavioral profile (Mellow et al., 2024). Previous studies have linked 24-h movement behaviors to indicators such as brain age, gray matter volume, and later risk of cognitive decline in older adults. This evidence base suggests that daily distribution of PA, SB, and sleep may describe cognitive aging processes more fully than isolated behavioral indicators, suggesting that the daily distribution of PA, SB, and sleep is relevant to cognitive aging (Balbim et al., 2025; Collins et al., 2025; Palazuelos-González et al., 2025). In the present guideline-adherence framework, the adherence count score therefore provides a practical summary of how multiple recommended behaviors accumulate within daily life. This interpretation should be understood as complementing behavior-specific evidence, rather than as a direct comparison between integrated and single-behavior approaches.

PA may be one important factor underlying this association. Intervention trials and review studies have reported improvements in executive function after aerobic exercise, resistance training, and multicomponent training in older adults, with inhibitory control often identified as a particularly responsive component (Fernandez-Gamez et al., 2026; Han et al., 2025; Lee et al., 2025b). This is consistent with the present findings, as older adults who met more guideline recommendations were more likely to maintain sufficient PA and showed better inhibitory control performance. Physiological findings provide additional support for this interpretation. Maintaining PA in later life has been linked to improved cardiorespiratory fitness, better macrovascular and microvascular cerebral perfusion, and stronger neurovascular coupling (Boa Sorte Silva et al., 2024; Li et al., 2025; Tari et al., 2025). PA may also promote plasticity in prefrontal and hippocampal networks and upregulate neuroplastic signaling pathways, including those involving brain-derived neurotrophic factor and insulin-like growth factor, thereby providing a biological basis for maintaining cognitive function (Behrad et al., 2024; Gholami et al., 2025). Viewed together, these literature-based pathways suggest that sustained PA may be relevant to brain function among older adults, although this biological interpretation remains hypothetical in the present study because no biological or neurophysiological markers were directly assessed.

SB and sleep also form core parts of the 24-h movement behavior framework. Excessive sedentary time does not only indicate insufficient PA. It may also reflect a behavioral pattern involving more low-energy-expenditure and low-cognitive-engagement sedentary activities among older adults. Higher SB has been related to poorer cognitive and brain health indicators in prior research. Even among older adults with relatively high PA levels, greater sedentary time remains associated with neurodegenerative changes and cognitive deterioration (Gogniat et al., 2025a, b). For specific sedentary behaviors, passive sedentary activities, particularly prolonged television viewing, have shown more consistent associations with dementia and adverse cognitive outcomes, whereas similar risks have not been observed for sedentary activities with greater cognitive engagement (Dejakaisaya et al., 2025; Wingood et al., 2024). In this sample, not meeting the SB recommendation may therefore indicate both longer sedentary exposure and fewer cognitively engaging behaviors during waking hours. Regarding sleep, growing evidence supports a nonlinear association between sleep and cognition, indicating that sleep duration at either end of the range is associated with less favorable cognitive trajectories. Insomnia and other sleep disorders have also been connected with poorer executive function, cognitive decline, and abnormal neuroimaging indicators (Carvalho et al., 2025; Zhang J. et al., 2025). Therefore, older adults who met more guideline recommendations may have been more likely to maintain both lower sedentary time and appropriate sleep duration. This pattern may reduce metabolic and inflammatory burden, support brain restoration, improve the allocation of daytime cognitive resources, and jointly contribute to better inhibitory control.

General self-efficacy also appeared to operate as an indirect pathway between meeting more guideline recommendations and inhibitory control. This finding suggests that 24-h movement behaviors may be related to inhibitory control not only through physiological pathways, but also through subjective psychological resources. In the cross-sectional model, general self-efficacy appeared as a modest psychological correlate that statistically accounted for part of the association between meeting more guideline recommendations and inhibitory control. This finding suggests that healthier movement behavior patterns, stronger self-efficacy beliefs, and better inhibitory control may co-occur within a broader self-regulatory profile in older adults. In prior work, self-efficacy has been tied to PA participation, persistence, and behavioral maintenance among older adults. Social support and outcome expectations may also interact with self-efficacy and form an important psychological basis for behavioral change (Safavi et al., 2025; Ying et al., 2025). From this perspective, when older adults are able to achieve exercise goals, reduce SB, and maintain relatively stable daily routines, they may accumulate mastery experiences and develop more positive evaluations of their own self-regulatory capacity. Higher general self-efficacy may then extend to cognitive task situations, helping older adults maintain task goals, inhibit irrelevant stimuli, and allocate attentional resources during conflict processing, thereby contributing to better inhibitory control performance.

However, the indirect contribution of general self-efficacy was modest, accounting for only 14.1% of the total effect. This indicates that general self-efficacy is a meaningful psychological correlate in the statistical model, but it is unlikely to be the dominant mechanism underlying the link between meeting more guideline recommendations and inhibitory control. This modest magnitude is consistent with prior meta-analytic evidence suggesting that self-efficacy is relevant to movement-related behaviors, but that the strength of this relationship varies by behavioral domain. For example, exercise self-efficacy has shown a moderate association with PA in older adults, whereas self-efficacy appears to have only a small association with sedentary behavior (Szczuka et al., 2021; Xie et al., 2025). In addition, recent meta-analytic evidence indicates that PA is associated with better late-life cognition, but that the overall association is generally weak, although still meaningful from a population health perspective (Iso-Markku et al., 2024). This modest indirect association may partly reflect how self-efficacy was measured. The present study assessed general self-efficacy rather than domain-specific self-efficacy related to PA, SB, or sleep management. Compared with efficacy beliefs in specific behavioral contexts, general self-efficacy has stronger cross-situational characteristics, but may provide relatively limited explanatory power for associations between specific health behaviors and specific cognitive tasks (Ghose et al., 2023; Luszczynska et al., 2005). Unmeasured physiological processes may also contribute to the remaining behavioral–cognitive association. These pathways include PA-related improvements in cerebral blood flow and enhanced plasticity in prefrontal and hippocampal networks, which may help delay cognitive aging (Behrad et al., 2024; Boa Sorte Silva et al., 2024; Gholami et al., 2025; Li et al., 2025). Sleep-related neural restoration processes may also be an important component of the pathway linking 24-h movement behaviors with inhibitory control (Zhang et al., 2024). Finally, other psychological mediators that are more proximal to cognitive outcomes may exist. For example, meeting a greater number of 24-h movement behavior guideline recommendations has been linked to lower anxiety and depressive symptoms, while depression is also significantly related to cognitive impairment among very old adults (Kolobaric et al., 2025; Tebar et al., 2024). Loneliness has also been associated with declines across multiple cognitive domains in older adults, whereas higher social participation is generally related to better cognitive and other health outcomes. These findings suggest that social participation-related factors may also be relevant to the association in older adults (Lee et al., 2025a; Zhang H. et al., 2025). Therefore, the current results position general self-efficacy as a secondary psychological pathway, rather than the main explanatory route, through which 24-h movement behaviors may be associated with inhibitory control.

Another notable result was that no participant met all three 24-h movement behavior guideline recommendations simultaneously. This result may be partly explained by the component-specific adherence pattern observed in the present sample. Although more than half of the participants met the PA recommendation, fewer participants met the SB and sleep recommendations, making simultaneous adherence to all three recommendations difficult. This pattern may reflect the daily routines of community-dwelling older adults in local residential settings, where regular walking or community-based activity may help some individuals achieve sufficient PA, whereas prolonged sedentary time and age-related changes in sleep routines may remain common challenges. This result suggests that, although the guidelines offer relatively attainable behavioral targets for older adults, achieving sufficient PA, reduced SB, and appropriate sleep duration at the same time remains difficult in real-world community settings. Although this finding is lower than that reported in some Chinese older adult samples using self-reported 24-h movement behavior data, it is not inconsistent with studies using objective measurement devices or studies involving older adults with chronic diseases. Evidence from Chinese older adult samples also indicates generally low guideline adherence, with only limited numbers of participants meeting all three recommendations (Liang et al., 2024; Luo et al., 2022).

Variation across studies may reflect differences in measurement methods, sample characteristics, sedentary time thresholds, and chronic disease burden. Present findings further suggest that health promotion for older adults should not focus only on increasing weekly moderate-to-vigorous PA, but should also address prolonged SB and sleep duration within an integrated 24-h movement behavior framework (Muñoz-Perete et al., 2025; Vásquez-Carrasco et al., 2025). Among older adults in particular, interventions targeting only one behavior may be insufficient to optimize overall daily time-use patterns. Future interventions should therefore integrate PA promotion, SB reduction, sleep management, and self-efficacy enhancement within a combined strategy to better support cognitive and psychological health in later life.

## 5 Strengths and limitations

This work has three main strengths. It focused on how 24-h movement behaviors relate to inhibitory control in older adults within an integrated 24-h movement behavior framework, rather than examining a single activity behavior or broad cognitive outcomes alone. Second, accelerometer-based PA and SB measures improved the objectivity of behavioral assessment. Third, after adjustment for multiple sociodemographic variables, this study examined both direct and mediating pathways, thereby providing evidence for the relationships among meeting more guideline recommendations, general self-efficacy, and inhibitory control.

Several caveats should be noted. Because of the cross-sectional design, causal relationships cannot be determined. Therefore, the indirect pathway through general self-efficacy should be interpreted as a cross-sectional statistical association rather than evidence of a causal mediation process. Reverse or alternative pathways are also possible. Given the relatively small observed mediating effect, longitudinal studies are needed to determine whether changes in 24-h movement behaviors precede changes in general self-efficacy and inhibitory control. Second, although the number of guideline recommendations met is a public health-relevant indicator, several limitations related to this operationalization and the present sample should be noted. The sample size was relatively limited, and no participant met all three guideline recommendations, which may restrict the generalizability of the findings. In addition, the present study used a count-based adherence score to indicate the number of recommendations met. This approach cannot fully capture the interdependent and compositional nature of 24-h movement behaviors. Moreover, this approach does not estimate time-reallocation effects or potential interactions among PA, SB, and sleep. Third, the conventional ActiGraph count-based cut-points used in this study were retained to maintain comparability with previous count-based studies. Because accelerometer cut-points may vary by wear location and population characteristics, the PA and SB estimates should be interpreted with this consideration in mind. Fourth, although the inclusion and exclusion criteria excluded participants with diagnosed severe neurological or psychiatric disorders, residual confounding related to depressive symptoms, chronic disease burden, medication use, sleep disorders, and baseline cognitive status may still exist. In addition, the lack of standardized baseline cognitive screening represents a methodological limitation of the present study. Therefore, subtle cognitive impairment or unmeasured clinical differences may have influenced the observed associations, which should be interpreted with caution in this respect.

Future studies using larger and more diverse older-adult samples should further examine the associations of 24-h movement behavior guideline adherence with general self-efficacy and cognitive outcomes. A feasible longitudinal approach would be to recruit community-dwelling older adults from multiple communities or senior centers and reassess 24-h movement behaviors, general self-efficacy, inhibitory control, and key clinical covariates at baseline, 12 months, and 24 months to clarify temporal ordering and reduce potential confounding. Specific adherence patterns across PA, SB, and sleep should also be considered, together with further refinement of accelerometer processing approaches, to provide a more detailed understanding of how 24-h movement behaviors relate to psychological and cognitive health in later life.

## 6 Conclusion

None of the participants in this study met all three 24-h movement behavior guideline recommendations, suggesting that, although these recommendations provide relatively attainable behavioral targets for older adults, achieving all of them simultaneously remains challenging in this population. Meeting more guideline recommendations was accompanied by better inhibitory control, and that general self-efficacy had a significant but modest partial mediating role in this association. For health promotion among older adults, future research should develop targeted interventions and further examine how 24-h movement behaviors are connected with cognitive and psychological outcomes, thereby providing more specific evidence for cognitive and psychological health promotion in later life.

## Data Availability

The datasets presented in this article are not readily available because the datasets are not publicly available because they contain sensitive health-related, behavioral monitoring, and cognitive task data from older adult participants. Access is restricted by ethical and privacy considerations. Requests to access the datasets should be directed to Lin Wang, wanglin123@126.com.

## Footnotes

1
https://schoemanna.shinyapps.io/mc_power_med/
