# Supplementary material for: Association of 24-hour movement guideline adherence with inhibitory control in older adults: the mediating role of general self-efficacy
Source: Front Psychol. 2026 Jul 22;17:1872136. doi: 10.3389/fpsyg.2026.1872136 (PMC13437439; doi:10.3389/fpsyg.2026.1872136)
Supplement: Supplementary file 1 [file Table_1.DOCX]

Supplementary Material

# 1 Supplementary Table S1

Supplementary Table S1. Subgroup descriptive statistics by educational level

| Variables | Primary school or below (n = 25) | Secondary school (n = 98) | Undergraduate degree or above (n = 87) |
| --- | --- | --- | --- |
| Age | 71.28 (6.00) | 71.31 (5.84) | 69.60 (6.51) |
| Sex |  |  |  |
| Male | 8 (32.0%) | 35 (35.7%) | 42 (48.3%) |
| Female | 17 (68.0%) | 63 (64.3%) | 45 (51.7%) |
| Number of guideline recommendations met |  |  |  |
| 0 | 2 (8.0%) | 11 (11.2%) | 10 (11.5%) |
| 1 | 17 (68.0%) | 60 (61.2%) | 52 (59.8%) |
| 2 | 6 (24.0%) | 27 (27.6%) | 25 (28.7%) |
| 3 | 0 (0.0%) | 0 (0.0%) | 0 (0.0%) |
